# Overcrowding in Emergency Department: Causes, Consequences, and Solutions—A Narrative Review

**DOI:** 10.3390/healthcare10091625

**Published:** 2022-08-25

**Authors:** Marina Sartini, Alessio Carbone, Alice Demartini, Luana Giribone, Martino Oliva, Anna Maria Spagnolo, Paolo Cremonesi, Francesco Canale, Maria Luisa Cristina

**Affiliations:** 1 Department of Health Sciences, University of Genova, Via Pastore 1, 16132 Genoa, Italy; 2 Operating Unit (S.S.D. U.O.) Hospital Hygiene, Galliera Hospital, Mura delle Cappuccine 14, 16128 Genoa, Italy; 3 Emergency Department, Galliera Hospital, Mura delle Cappuccine 14, 16128 Genoa, Italy; 4 Medical Service Management, Galliera Hospital, Mura delle Cappuccine 14, 16128 Genoa, Italy

**Keywords:** overcrowding, emergency department, hospital admission

## Abstract

Overcrowding in Emergency Departments (EDs) is a phenomenon that is now widespread globally and causes a significant negative impact that goes on to affect the entire hospital. This contributes to a number of consequences that can affect both the number of resources available and the quality of care. Overcrowding is due to a number of factors that in most cases lead to an increase in the number of people within the ED, an increase in mortality and morbidity, and a decrease in the ability to provide critical services in a timely manner to patients suffering from medical emergencies. This phenomenon results in the Emergency Department reaching, and in some cases exceeding, its optimal capacity. In this review, the main causes and consequences involving this phenomenon were collected, including the effect caused by the SARS-CoV-2 virus in recent years. Finally, special attention was paid to the main operational strategies that have been developed over the years, strategies that can be applied both at the ED level (microlevel strategies) and at the hospital level (macrolevel strategies).

## 1. Introduction

The Emergency Department (ED) is one of the most crowded hospital units, where many patients with various medical conditions, including high-risk patients, are admitted [1]. The main purpose of the ED is to treat emergency and urgent cases that need immediate assistance through a rapid diagnosis and the administration of a medical or surgical treatment in a very short time. It has now been established that the malfunctioning of health services in the community leads to improper access to the ED, especially in the geriatric and pediatric age groups [1,2,3]. ED’s crowding, sometimes referred to as overcrowding, has been identified as a problem for a timely and efficient assistance since the 1980s [4].

Overcrowding can be defined as a situation in which the performance of the emergency department is compromised, mainly due to the excessive number of patients waiting for consultation, diagnosis, treatment, transfer, or discharge [2,5]; overcrowding is characterized by an imbalance between supply and demand [2].

Although many factors contribute to overcrowding, the latter depends essentially on three factors: the incoming volume of patients (input), the time to process and treat patients (throughput), and the volume of patients leaving the ED (output) [6].

Among the different factors, patient boarding was found to be one of the most significant [7]. Boarding is the practice of keeping patients admitted to the ED for prolonged periods due to inadequate capacity of inpatient wards [7,8]. Boarding, and overcrowding in general, has negative effects on patient care, mortality, morbidity, patient satisfaction, and quality of care [4,9,10]. These also contribute to a longer length of stay (LOS) in the ED, an increased rate of patients leaving the ED without being seen (LWBS, left without being seen), and increased medical errors [11,12,13].

ED overcrowding has turned into a serious health problem, as the number of EDs is decreasing, while the number of patients requiring emergency services is increasing [11,13]. It has been reported in the literature that overcrowding occurs most often in EDs with an annual volume of over 40,000 visits [11,14].

An accurate measurement of crowding in the ED and an evidence-based understanding of its impact are essential prerequisites before attempting to find solutions [6]. Although there are various scores for estimating the different degrees of overcrowding, to date, there is still no gold standard for measuring this phenomenon [4,15]. A review in the literature suggests that overcrowding is defined by the following three estimation indices: National Emergency Department Overcrowding Score (NEDOCS), Community Emergency Department Overcrowding Score (CEDOCS), and Severely-overcrowded-Overcrowded and Not-overcrowded Estimation Tool (SONET). The most frequently used score is the NEDOCS, developed by Weiss and colleagues [15]; NEDOCS converts a series of variables into a score, which is related to the degree of overcrowding perceived by the professionals performing their tasks at that moment. The scale has a range between 0 and 200 points, where a rating of 101 or more indicates a condition of overcrowding [16].

Finally, among the measurement systems that can be evaluated to estimate overcrowding, we also have ED occupancy, ED length of stay, ED volume, ED boarding time, number of boarders, waiting room number, and the Emergency Department Work Index (EDWIN) score. So, in order to develop efficient solutions to overcrowding, it is essential not only to understand its various causes and effects but also to estimate its actual impact on the health care system [4].

This paper aims to make an additional contribution to the understanding of overcrowding in the ED by providing an analytical overview of the causes, effects, and solutions to the problem; to our knowledge, there are not many papers that deal with the topic with this organic vision.

## 2. Materials and Methods

In this narrative review article, a comprehensive search on PubMed.gov, Scopus, ISI Web of Science, Science Direct, and Google Scholar using as keywords “Overcrowding”, “Emergency Department”, “Hospital admission”, “Length of Stay”, “Waiting time”, and “inpatient boarding” up to June 2022 using Medical Subject Headings (MeSH) terms as vocabulary was performed. Inclusion criteria were: (1) research articles with quantitative details and information on the relationship between the causes that lead to overcrowding in Emergency Departments and the consequences that this phenomenon entails; (2) articles describing possible strategies already adopted or adoptable in the future to address the effect that overcrowding has on the Emergency Department were considered. Exclusion criteria were articles not directly pertinent to the query string or articles not containing sufficient information on the relationship between overcrowding and Emergency Departments (Table 1).

Two authors were involved during the screening of the literature. Articles were firstly selected based on title and abstract. The full text of relevant research was then acquired and assessed. Each reference of the selected articles was checked in order not to miss any relevant article. The authors independently read all the papers. A complete consensus was achieved through discussion for the texts included in this study.

## 3. Results

After removal of duplicate items, the resulting list comprised 113 nonredundant articles, and 61 were finally considered in our narrative review.

### 3.1. Causes of ED Overcrowding

As anticipated, the problem of overcrowding in EDs can be due to multiple factors, which may be represented by the input–throughput–output model (Table 2). Overcrowding is a multifactorial and complex phenomenon; these different factors are independent from one another but are closely connected and influenced by additional factors [10,17,18].

#### 3.1.1. Input Factors

Input factors are those which lead to increased admissions in the ED [10,19]. These include the development of new or unsatisfied care needs in appropriate areas of community care, the progressive aging of the population, the increasing number of complex patients, the introduction of new diagnostic and treatment technologies, and the increase in admissions for diseases related to seasonal epidemiology (e.g., flu epidemics and heat waves) or related to time of year/week. Input factors cannot be controlled by the ED [10,20].

It has been observed that the number of escorts has a negative impact on the workflow in the ED. Although they do not play an active role in the process, they may unintentionally annoy the staff and consequently increase the workload and pressure in the ED. For this reason, some hospitals in Israel have reduced the number of escorts per patient to one [18].

Another important input factor is avoidable accesses, which can be considered “conditions susceptible of ambulatory treatment”, including major chronic diseases, double accesses related to inappropriate performance of the territorial emergency network, more generally repeated accesses (e.g., frequent flyers) as well as all improper accesses related to territorial organizational patterns (e.g., schedules) and patient habits [20,21].

Some studies have found a positive relation between ED overcrowding and patient admission rates, and this effect further increases for patients classified as less severe. These findings suggest that ED overcrowding could be causing multiple problems, such as unnecessary consumption of medical resources due to unnecessary hospitalizations [22,23].

#### 3.1.2. Throughput Factors

Throughput factors are those internal to the ED itself and which affect the time from patient admission to discharge, hospitalization, or transfer (LOS) [10].

Among the throughput factors, the one that most influences a patient’s LOS is the need for specialistic consultations and/or additional instrumental diagnostic investigations, procedures that are increasingly necessary both because of the increasing average age of patients and comorbidities and to ensure appropriate hospitalizations and safe discharges [20]. Prolonged inpatient times may be the result of overcrowding, delayed radiological and laboratory test results, delayed and inappropriate consultations, and inadequate number of inpatient beds [21].

ED productivity is also affected by the work efficiency of hospital staff. It is important that the demand for assistance and the actual working capacity are balanced so that it can be ensured that the flow goes on properly, especially under emergency conditions [20]. Anything that compromises the flow of patients through the ED can lead to overcrowding [19]; if a resource (e.g., medical staff, consultant, diagnostic service, or bed slot) has a demand that exceeds its capacity, there will be a blockage in the system; therefore, the flow will be regular if available resources balance the demand in all the stages of the path [6,20].

#### 3.1.3. Output Factors

Output factors can be summarized by the failure to transfer patients out of the ED following all necessary treatment. Among these factors are the availability of beds and the delay in transporting patients to free up space in the ED, thus leading patients to remain waiting both to reach the appropriate department and for their eventual discharge. So, it is clear just how large the impact is, which these factors exert on overcrowding, given that they burden not only the level of space and bed availability, but also other health care resources [10].

As mentioned earlier, bed availability and inability to receive adequate home care are among the most important factors causing overcrowding. These not only affect EDs locally but also globally, and this also determines other phenomena of exit block and boarding [10,20].

Exit block is a phenomenon that is likely to occur when patients in the ED are unable to access beds in a reasonable time. The result is an increase in overcrowding, since in these cases the hospital, and especially the ED, has already reached maximum limits of admission, and new arrivals will lead to their waiting longer than the necessary time.

Exit block leads to important consequences, both in terms of patient health, increased waiting time, boarding, and quality of care. Many studies have focused on the negative impact of exit block, not only on low-risk patients but also on those in need of immediate surgery, for example, in emergency situations. Finally, it has been observed how this phenomenon and overcrowding can affect a patient’s choice to leave the ED, without first undergoing a proper medical examination, potentially leading to a worse outcome [10].

A lack of beds can lead to the practice of retaining patients within the ED thus leading to the phenomenon of boarding, which is directly dependent on the exit block. Indeed, like the latter, boarding has among its main consequences the exceeding of the levels of care that can be guaranteed by the hospital. Studies have shown that in some large EDs, at least 40% of the health care staff spend their time on patients who have already gone through a medical consultation but are unable to leave the ED due to the above-mentioned phenomena, rather than taking care of patients in the wards [10].

#### 3.1.4. The Impact of SARS-CoV-2 on ED Overcrowding

Over the past two years, hospitals have faced difficulties brought by the SARS-CoV-2 pandemic, and the effects of the latter on the availability of emergency services and ED overcrowding are still poorly evaluated [24].

The pandemic has been a challenge for the ED [1,25] in several aspects, and this has brought changes in the management of staff, patients, and wards. Indeed, potentially infected patients must be separated from others; staff must wear protective clothing that limits productivity, and vital parameters must be monitored more frequently. There is a high risk that this increased workload could result in crowding of the ED [26,27].

It can be observed that the waiting time for hospitalization has lengthened, partly because of the need to screen all patients before assigning them to a “clean” ward or a COVID unit, in order to ensure that positive patients, even if asymptomatic, were not admitted to clean wards contributing to the spread of the virus [10].

The increasing crowding during the pandemic is believed to be due mainly to three factors: the mismatch between the need for intensive care unit beds and the number of available beds, the large number of frail patients requiring stabilization before admission to hospital wards, and the change in management of all patients [24]. Therefore, ED overcrowding has been a direct consequence of hospital overcrowding in general [10].

As a result of these two years, it has been noticed that measures are therefore needed to alleviate crowding and reduce exit block, so that hospitals are prepared to respond adequately to any future pandemics [24].

### 3.2. Effects and Consequences of Overcrowding in EDs

The most evident effect of overcrowding in the performance of an ED is an increase in patient waiting time; this increase causes an increment in the number of patients leaving the ED before being visited by a physician, which is defined as left without being seen (LWBS); however, it has been observed that this group of patients complains of a progressive worsening of health conditions and returns shortly afterwards to be hospitalized (return visit). Several studies have found that the quality of treatment in overcrowded situations worsens significantly; it has been shown that in patients with myocardial infarction, an increase in door-to-needle time, the time between patient evaluation and drug administration, was significantly longer in overcrowded situations compared to normal timing [2]. An Australian retrospective study showed a clear increase in mortality of patients admitted to the ED during an overcrowded shift compared with those admitted during a normal shift. The authors of this study calculated that there are 13 deaths per year in their hospital due to overcrowding in the ED [28].

Overcrowding reduces ED capacity, affects quality of care, increases the risk of adverse outcomes for patients, especially cardiac and intubated patients, and increases the risk of hospital-acquired infections and the likelihood of patient management errors [29,30].

ED staff also suffer the effects of overcrowding; job satisfaction is affected by these stressful situations, and overcrowding has been identified as a major reason for staff reduction [2,31].

The potential financial impact of overcrowding is not insignificant; in fact, the resulting increase in reconsultations and hospitalizations, worse quality of treatment, dissatisfaction of health care staff, and morbidity lead to higher treatment costs [2,32]. According to a study, boarding increases the cost by USD 6.8 million over 3 years. Reducing boarding time by just one hour would increase revenue by USD 13,298 per day or USD 4.9 million per year [4,33,34].

Return visit (RV) is often used as a quality indicator for ED because it can be caused by premature discharge, missed diagnosis, or failure of treatment or discharge planning [35]. RVs not only delay adequate treatment of patients, but also increase resource use and medical costs [35,36]. Other factors, such as disease progression, lack of improvement, or patient concern and fear about their condition, contribute to this problem. Overcrowding is a health problem worldwide that leads to an increase in misdiagnoses and medical errors [35,37]. ED staff must always provide timely care to urgent patients; therefore, when the ED is overcrowded, physicians accelerate the patient discharge process to prepare an empty bed for new patients [2].

### 3.3. Solution to Overcrowding

Regarding the resolution of overcrowding, several actions are needed, not only at the medical level but also at the bureaucratic level. These can be divided into two levels that act in synergy: microlevel and macrolevel strategies [4,10] as shown schematically in Table 3.

#### 3.3.1. Microlevel Strategies

Microlevel strategies are designed to fight the problem of overcrowding and boarding and include those changes that can be applied at the level of the ED [4].

##### Acceleration of Diagnostic Pathways

The use of standardized diagnostic pathways can be extremely useful in the standardizing care process, diagnosis, and treatment in order to reduce waiting times, the chance of error and, in some circumstances, hospitalization rates. They are also crucial in enhancing outcomes by reducing adverse events and mortality [4,10].

One of the possible strategies is to introduce point-of-care procedures (POCTs) in EDs. Internal POCTs in EDs offer several advantages over the determination of laboratory parameters that would normally be conducted in a central laboratory. This reduces sample transport times and the communication of results from the central laboratory to the receiver [2,5]. There are promising data in this regard; a recent U.S. study demonstrated a 1 h reduction in average treatment time through the use of point-of-care laboratory testing in triage [38].

##### Fast Track

To accelerate the treatment of nonurgent patients with less serious symptoms or illnesses (green and white codes), an alternative pathway, the so-called “fast track,” has already been introduced in many EDs. The fast track consists of direct transfer from triage to a specialist physician. Numerous studies have shown that the introduction of this accelerated pathway has brought several benefits, not only in terms of reducing the waiting time and treatment of patients but also in reducing the number of patients who left the ED before being seen (LWBS) by a physician [2,3,39].

##### Outpatient Services outside the ED

Another microlevel strategy is to redirect patients accessing the ED to alternative health care resources by making special reference to outpatient services; in fact, on some occasions, patients primarily access the ED because they are unable to find their way around the health care system. This phenomenon is more prevalent among certain social groups, such as low social classes, low literacy levels, and patients who fear the stigma and shame associated with certain conditions [5,40,41]. Imaging techniques for noncritical patients could be taken over by other adequate facilities in order to give priority and ensure access to emergency diagnostic procedures for critical patients.

##### Setting Home Care

Another way that can be taken to reduce overcrowding in EDs is home care. After appropriate initial diagnosis and stabilization of the patient, for those who do not require hospitalization, home care can play a key role in the continuation of care.

So, home care brings benefits not only in terms of reducing overcrowding but also in terms of quality of care and patient satisfaction to different categories of patients. These benefits have been highlighted especially in the elderly, who find that being able to continue treatment in a familiar and comfortable environment benefits their health [4,10].

##### Team Triage

Team triage refers to the triage of patients performed by nurses in conjunction with physicians. However, some studies have shown conflicting data to date [2]; some of these found a mortality benefit, but no effect on waiting or treatment time [2]. In contrast, other studies showed significantly lower treatment time [42].

Another intervention on triage that has been promising is to give nurses more authority, for example, by giving them the ability to request diagnostic tests, such as X-rays, even before the physician has examined the patient [5,39,43]. However, it is highly recommended that nurses acquire adequate training before assuming this additional role [26].

Furthermore, in a study by Debono et al. [44], it was demonstrated that medical or nursing staff trained to conduct a telephone triage system could decrease the number of accesses in a pediatric ED, and this possible solution could be extended to other age groups as well.

##### Artificial Intelligence (AI) and Machine Learning

AI and machine learning represent a new approach to implement the most effective strategies to combat the problem of overcrowding. Cabezuelo studied the best set of variables that explain the phenomenon of the return of patients to the emergency department of a hospital in less than 72 h. He found that the best machine learning algorithm is a neural network [45]. Arnaud et al. studied the early prediction of patient hospitalization at the triage stage applying data analytics [46].

#### 3.3.2. Macrolevel Strategies

Macrolevel strategies can be put into practice to fight the problem of overcrowding similarly to microlevel strategies, but, unlike the latter, they are applied at the hospital and/or care system level [4,10].

##### Simplifying the Admission Process

Simplifying the admission processes could provide better control of patient flows by reducing waiting times and ensuring better management of overcrowding in EDs [10,47].

Verbal handover between two attending physicians is still the most effective method to ensure safe and smooth transitions; however, in periods of rapid patient influx or in academic institutions where students serve as the primary workforce, this can be difficult. The goal would be to have a standardized admission process used by all inpatient services in order to reduce delays and potentially maximize hospital service performance.

In hospitals with the capability, a standardized electronic signature process could enable more efficient and asynchronous admission [4,48].

##### Reverse Triage

Reverse triage is a process to identify hospitalized patients who are stable and do not require further treatment and can therefore be discharged without any risk [10,49].

Early discharge from the hospital is also facilitated and supported by cooperation with external facilities, such as hospices, nursing homes, rehabilitation centers, and the patients’ own homes, of course with a proper support program if necessary [10,49]. The addition of a 24–48 h postdischarge telemedicine follow-up period, together with reverse triage and early discharge processes, can potentially facilitate both caregivers and patients by thereby promoting the availability of hospital beds for new admissions [4,50].

##### Smoothing Elective Admissions

Although variability in the number of hospitalizations in emergency medicine cannot be controlled, studies over time have demonstrated that it is highly predictable based on weather, season, and epidemiology [51,52]. The remaining hospital admissions are elective scheduled admissions, which typically are scheduled at the beginning of each week and have been shown to have a deeply negative impact on overall flow and boarding.

The problematic aspect is related to the fact that elective hospitalizations often compete with urgent hospitalizations related to ED admissions.

Much work has been done on elective scheduling of surgical hospitalizations, and this has led to a substantial decrease in boarding and improved bed availability in the inpatient and intensive care units. Because of the peaks in elective hospitalizations at the beginning of the week, spreading them evenly over the week would improve the hospital’s bed capacity [51,53,54].

##### Early Discharge

Without the early discharge of hospitalized patients, new patients admitted to the ED are at risk of experiencing boarding. According to a study by Powell et al., to contrast this issue, early discharge before noon has been shown to improve ED flow, reducing boarding by 96% [51,55]. It was also found that at New York University, increasing the number of patients discharged before noon led to an overall reduction in length of stay. Their efforts were guided by the finding that hospitalized patients arriving at the inpatient unit before noon had an average length of stay of 0.6 days less than those arriving after noon [17].

##### Weekend Discharge

On weekends, the number of discharges is usually nearly 50% lower than the number of discharges on weekdays [51]. The increase in weekend discharges can substantially increase bed availability earlier in the week and reduce the hospital’s overall LOS. Although this may require resources that are often unavailable on weekends, such as echographies, MRIs, and stress tests, increased weekend services result in less demand for them during the week. For this reason, it is not necessary to increase staffing, but it is sufficient to redistribute some of it on weekends [17,56].

##### Full Capacity Protocol or Action Plan

Financial demands require hospitals to operate at nearly full capacity, but when the capacity is exhausted, hospitals should use a program to manage excess hospitalized patients and reduce boarding in the ED, such as the full capacity protocol (FCP) that consists of transferring patients from the hallways of the ED to the hallways of inpatient wards.

The establishment of an FCP has been studied extensively in different settings and has been shown to reduce waiting time and boarding, improve productivity, reduce overall length of stay, and improve patient satisfaction [17,51,57].

##### Legislation and Guidelines

The awareness of the overcrowding problem by members of hospital management is a key aspect that must be approached to solve the problem [10,49].

In case there is no improvement, despite the possibility of making structural and organizational changes that could reduce the problem, it is necessary to enhance regulations and draft stronger legislation to regulate overcrowding, through effective and precise guidelines, in order to solve the issue at a higher level [7,10].

#### 3.3.3. Observation Unit

Other strategies that could have a positive impact on hospital admissions are observation units (OBIs—units of short and intensive observation), which are a link between microlevel and macrolevel factors, as they are located at the intersection of ED and hospital care [4,10,58,59].

Patients who may benefit from the presence of an observation unit are those who, after receiving a diagnosis or starting a treatment, do not require prolonged hospitalization but need to be kept under observation for a short period of time. Thus, the institution of OBIs could reduce overcrowding in EDs, while allowing continuous monitoring and treatment of patients.

An Italian research group showed that over the years of operation of an OBI team, a stabilization of the phenomena of “boarding” and “exit block” was observed, despite an increase in the number of ED admissions and the need for hospitalization of the patients themselves. A containment of the length of stay and improvement in the outcomes of some categories of patients was also observed [10]; these results are in line with data from other European and American research groups [10,60,61].

## 4. Discussion

Considering the growing importance of overcrowding in EDs and its potential effects on the wellness of patients and employees, the need to develop strategies to deal with or mitigate the problem has become evident [2]. As has been described, the causes leading to overcrowding in EDs are multiple, starting with input causes and ending with output causes. Only knowledge and awareness of the issue can lead us to put in place the most appropriate strategies to be able to counteract the problem and bring it under control. In this regard, this review was conducted, starting with an analysis of the causes and consequences, and then focusing mainly on the strategies that can be used to counteract this phenomenon.

This review to our knowledge presents a detailed analysis of possible solutions to overcrowding not reported in other reviews. It also presents a summary of the main indicators of overcrowding although there is currently no gold standard.

There are several limitations in this narrative review. First, only articles in English were included, and therefore important information published in other languages may be missing, as this is a worldwide issue. The studies considered included pediatric EDs in some cases, with their specific issues, in other Emergency Departments aimed at the general population.

## 5. Conclusions

In this regard, numerous strategies have been collected and proposed in order to be implemented both at the ED level (microlevel strategies) and at the hospital level (macrolevel strategies). The goal should be to carry out an approach that takes into consideration not just the ED but also the hospital, the health care system in general, and the community.

## Figures and Tables

**Table 1:** Search strategy.

Search Strategy	Details
Search string	(Emergency Department [MeSH Terms]) AND (Overcrowding) OR (Crowding)(Overcrowding) AND (ED) OR (Emergency Department)
Databases	PubMed/MEDLINE, Scopus, Cochrane, and Google Scholar
Inclusion criteria	(1) research articles with quantitative details and information on the relationship between the causes that lead to overcrowding in Emergency Departments and the consequences that this phenomenon entails; (2) articles describing possible strategies already adopted or adoptable in the future to address the effect that overcrowding has on the Emergency Department were considered; (3) all kinds of study designs and reviews
Exclusion criteria	Items not directly pertinent to the query string and articles not containing sufficient information on the relationship between Overcrowding and Emergency Department Study design: editorial, commentaries, expert opinions, letters to editor, and abstracts
Time filter	None (from inception)
Language filter	Only Italian and English articles

**Table 2:** Main causes of overcrowding.

Factors	Causes
**Input** *due to the volume of patients arriving and waiting to be seen*	Presentations with more urgent and complex care needs • Emergencies
	Increase in presentations by the elderly
	High volume of low-acuity presentations (LAPs)
	Access to primary care • The poor and uninsured who lack primary care
	Limited access to diagnostic services in community • The malfunctioning of health care services in the community
	Inappropriate use of emergency services • Unnecessary visits • “Frequent flyer” patients • Nonurgent visits • The majority of ED incomings resulted from self-referral process
	The number of escorts accompanying a patient
**Throughput** *due to the time to process and/or treat patients*	ED nursing staff shortages Low staffing and resource levels
	Presence of junior medical staff in ED
	Delays in receiving test results and delayed disposition decisions
	Number of tests (blood test and urinalysis) required to be performed per patient
	Too long a consultation time
	Patient degree of gravity
	Bed availability (both in the ED and in the hospital)
**Output** *due to the volume of patients leaving the ED*	Boarding
	Exit block
	Lack of available hospital beds
	Inefficient planning of discharging patients
**Others**	An increase in closures of a significant number of EDs
	Time of the year • Influenza season • Seasonal illness
	Weekend, holiday periods
	COVID-19

**Table 3:** Microlevel and macrolevel strategies.

Strategies	Solutions
**Microlevel strategies** *applied at the level of the Emergency Department*	Acceleration of diagnostic pathways
	Fast track
	Outpatient services outside the ED
	Setting home care
	Observation unit
	Team triage Artificial intelligence (AI) and machine learning
**Macrolevel strategies** *applied at the hospital and/or care system level*	Simplifying the admission process
	Reverse triage
	Smoothing elective admissions
	Early discharge
	Weekend discharge
	Full capacity protocol or action plan
	Legislation and guidelines

## References

[1] Babatabar-Darzi H., Jafari-Iraqi I., Mahmoudi H., Ebadi A. (2020). Overcrowding Management and Patient Safety: An Application of the Stabilization Model. Iran. J. Nurs. Midwifery Res..

[2] Lindner G., Woitok B.K. (2021). Emergency Department Overcrowding: Analysis and Strategies to Manage an International Phenomenon. Wien. Klin. Wochenschr..

[3] Adriani L., Dall’Oglio I., Brusco C., Gawronski O., Piga S., Reale A., Buonomo E., Cerone G., Palombi L., Raponi M. (2022). Reduction of Waiting Times and Patients Leaving Without Being Seen in the Tertiary Pediatric Emergency Department: A Comparative Observational Study. Pediatr. Emerg. Care.

[4] Kenny J.F., Chang B.C., Hemmert K.C. (2020). Factors Affecting Emergency Department Crowding. Emerg. Med. Clin. N. Am..

[5] Yarmohammadian M., Rezaei F., Haghshenas A., Tavakoli N. (2017). Overcrowding in Emergency Departments: A Review of Strategies to Decrease Future Challenges. J. Res. Med. Sci..

[6] Badr S., Nyce A., Awan T., Cortes D., Mowdawalla C., Rachoin J.-S. (2022). Measures of Emergency Department Crowding, a Systematic Review. How to Make Sense of a Long List. Open Access Emerg. Med..

[7] Rabin E., Kocher K., McClelland M., Pines J., Hwang U., Rathlev N., Asplin B., Trueger N.S., Weber E. (2012). Solutions To Emergency Department ‘Boarding’ And Crowding Are Underused And May Need To Be Legislated. Health Aff..

[8] American College of Emergency Physicians (2011). Practice Guideline. Definition of Boarded Patient. Ann. Emerg. Med..

[9] Emergency Medicine Practice Committee (2016). Emergency Department Crowding: High Impact Solutions. https://www.acep.org/globalassets/sites/acep/media/crowding/empc_crowding-ip_092016.pdf.

[10] Savioli G., Ceresa I.F., Gri N., Bavestrello Piccini G., Longhitano Y., Zanza C., Piccioni A., Esposito C., Ricevuti G., Bressan M.A. (2022). Emergency Department Overcrowding: Understanding the Factors to Find Corresponding Solutions. J. Pers. Med..

[11] Phillips J.L., Jackson B.E., Fagan E.L., Arze S.E., Major B., Zenarosa N.R., Wang H. (2017). Overcrowding and Its Association With Patient Outcomes in a Median-Low Volume Emergency Department. J. Clin. Med. Res..

[12] Epstein S.K., Huckins D.S., Liu S.W., Pallin D.J., Sullivan A.F., Lipton R.I., Camargo C.A. (2012). Emergency Department Crowding and Risk of Preventable Medical Errors. Intern. Emerg. Med..

[13] Carter E.J., Pouch S.M., Larson E.L. (2014). The Relationship Between Emergency Department Crowding and Patient Outcomes: A Systematic Review: Emergency Department Crowding and Patient Outcomes. J. Nurs. Scholarsh..

[14] Welch S.J., Augustine J.J., Dong L., Savitz L.A., Snow G., James B.C. (2012). Volume-Related Differences in Emergency Department Performance. Jt. Comm. J. Qual. Patient Saf..

[15] Asaro P.V., Lewis L.M., Boxerman S.B. (2007). Emergency Department Overcrowding: Analysis of the Factors of Renege Rate. Acad. Emerg. Med..

[16] Boldori H.M., Ciconet R.M., Viegas K., Schaefer R., dos Santos M.N. (2021). Cross-Cultural Adaptation of the Scale National Emergency Department Overcrowding Score (NEDOCS) for Use in Brazil. Rev. Gaúcha De Enferm..

[17] Salway R., Valenzuela R., Shoenberger J., Mallon W., Viccellio A. (2017). Emergency department (ed) overcrowding: Evidence-based answers to frequently asked questions. Rev. Médica Clínica Las Condes.

[18] Wachtel G., Elalouf A. (2020). Addressing Overcrowding in an Emergency Department: An Approach for Identifying and Treating Influential Factors and a Real-Life Application. Isr. J. Health Policy Res..

[19] Affleck A., Parks P., Drummond A., Rowe B.H., Ovens H.J. (2013). Emergency Department Overcrowding and Access Block. CJEM.

[20] Ministero Della Salute Linee di Indirizzo Nazionali per lo Sviluppo del Piano di Gestione del Sovraffollamento in Pronto soccorso. https://www.salute.gov.it/imgs/C_17_pubblicazioni_3143_allegato.pdf.

[21] Erenler A.K., Akbulut S., Guzel M., Cetinkaya H., Karaca A., Turkoz B., Baydin A. (2014). Reasons for Overcrowding in the Emergency Department: Experiences and Suggestions of an Education and Research Hospital. Turk. J. Emerg. Med..

[22] Jung H.M., Kim M.J., Kim J.H., Park Y.S., Chung H.S., Chung S.P., Lee J.H. (2021). The Effect of Overcrowding in Emergency Departments on the Admission Rate According to the Emergency Triage Level. PLoS ONE.

[23] Chen W., Linthicum B., Argon N.T., Bohrmann T., Lopiano K., Mehrotra A., Travers D., Ziya S. (2020). The Effects of Emergency Department Crowding on Triage and Hospital Admission Decisions. Am. J. Emerg. Med..

[24] Savioli G., Ceresa I.F., Novelli V., Ricevuti G., Bressan M.A., Oddone E. (2022). How the Coronavirus Disease 2019 Pandemic Changed the Patterns of Healthcare Utilization by Geriatric Patients and the Crowding: A Call to Action for Effective Solutions to the Access Block. Intern. Emerg. Med..

[25] Al-Surimi K., Yenugadhati N., Shaheen N., Althagafi M., Alsalamah M. (2021). Epidemiology of Frequent Visits to the Emergency Department at a Tertiary Care Hospital in Saudi Arabia: Rate, Visitors’ Characteristics, and Associated Factors. Int. J. Gen. Med..

[26] Bittencourt R.J., Stevanato A.D.M., Bragança C.T.N.M., Gottems L.B.D., O’Dwyer G. (2020). Interventions in Overcrowding of Emergency Departments: An Overview of Systematic Reviews. Rev. De Saúde Pública.

[27] af Ugglas B., Skyttberg N., Wladis A., Djärv T., Holzmann M.J. (2020). Emergency Department Crowding and Hospital Transformation during COVID-19, a Retrospective, Descriptive Study of a University Hospital in Stockholm, Sweden. Scand. J. Trauma Resusc. Emerg. Med..

[28] Richardson D.B. (2006). Increase in Patient Mortality at 10 Days Associated with Emergency Department Overcrowding. Med. J. Aust..

[29] Menon N.V.B., Jayashree M., Nallasamy K., Angurana S.K., Bansal A. (2021). Bed Utilization and Overcrowding in a High-Volume Tertiary Level Pediatric Emergency Department. Indian Pediatr..

[30] Jo S., Jeong T., Jin Y.H., Lee J.B., Yoon J., Park B. (2015). ED Crowding Is Associated with Inpatient Mortality among Critically Ill Patients Admitted via the ED: Post Hoc Analysis from a Retrospective Study. Am. J. Emerg. Med..

[31] Crook H.D., Taylor D.M., Pallant J.F., Cameron P.A. (2004). Workplace Factors Leading to Planned Reduction of Clinical Work among Emergency Physicians. Emerg. Med..

[32] Green D., Ruel J. (2020). Impact of Advanced Practice Prehospital Programs on Health Care Costs and ED Overcrowding: A Literature Review. Adv. Emerg. Nurs. J..

[33] Krochmal P., Riley T.A. (1994). Increased Health Care Costs Associated with ED Overcrowding. Am. J. Emerg. Med..

[34] Pines J.M., Batt R.J., Hilton J.A., Terwiesch C. (2011). The Financial Consequences of Lost Demand and Reducing Boarding in Hospital Emergency Departments. Ann. Emerg. Med..

[35] Kim D., Park Y.S., Park J.M., Brown N.J., Chu K., Lee J.H., Kim J.H., Kim M.J. (2020). Influence of Overcrowding in the Emergency Department on Return Visit within 72 H. J. Clin. Med..

[36] Duseja R., Bardach N.S., Lin G.A., Yazdany J., Dean M.L., Clay T.H., Boscardin W.J., Dudley R.A. (2015). Revisit Rates and Associated Costs After an Emergency Department Encounter: A Multistate Analysis. Ann. Intern. Med..

[37] Di Somma S., Paladino L., Vaughan L., Lalle I., Magrini L., Magnanti M. (2015). Overcrowding in Emergency Department: An International Issue. Intern. Emerg. Med..

[38] Singer A.J., Taylor M., LeBlanc D., Meyers K., Perez K., Thode H.C., Pines J.M. (2018). Early Point-of-Care Testing at Triage Reduces Care Time in Stable Adult Emergency Department Patients. J. Emerg. Med..

[39] Oredsson S., Jonsson H., Rognes J., Lind L., Göransson K.E., Ehrenberg A., Asplund K., Castrén M., Farrohknia N. (2011). A Systematic Review of Triage-Related Interventions to Improve Patient Flow in Emergency Departments. Scand. J. Trauma Resusc. Emerg. Med..

[40] Savioli G., Ceresa I.F., Maggioni P., Lava M., Ricevuti G., Manzoni F., Oddone E., Bressan M.A. (2020). Impact of ED Organization with a Holding Area and a Dedicated Team on the Adherence to International Guidelines for Patients with Acute Pulmonary Embolism: Experience of an Emergency Department Organized in Areas of Intensity of Care. Medicines.

[41] Lee I.-H., Chen C.-T., Lee Y.-T., Hsu Y.-S., Lu C.-L., Huang H.-H., Hsu T.-F., How C.-K., Yen D.H.-T., Yang U.-C. (2017). A New Strategy for Emergency Department Crowding: High-Turnover Utility Bed Intervention. J. Chin. Med. Assoc..

[42] Burström L., Nordberg M., Örnung G., Castrén M., Wiklund T., Engström M.-L., Enlund M. (2012). Physician-Led Team Triage Based on Lean Principles May Be Superior for Efficiency and Quality? A Comparison of Three Emergency Departments with Different Triage Models. Scand. J. Trauma Resusc. Emerg. Med..

[43] Jeyaraman M.M., Copstein L., Al-Yousif N., Alder R.N., Kirkland S.W., Al-Yousif Y., Suss R., Zarychanski R., Doupe M.B., Berthelot S. (2021). Interventions and Strategies Involving Primary Healthcare Professionals to Manage Emergency Department Overcrowding: A Scoping Review. BMJ Open.

[44] Debono P., Debattista J., Attard-Montalto S., Pace D. (2012). Adequacy of Pediatric Triage. Disaster Med. Public Health Prep..

[45] Sarasa Cabezuelo A. (2020). Application of Machine Learning Techniques to Analyze Patient Returns to the Emergency Department. J. Pers. Med..

[46] Arnaud E., Elbattah M., Gignon M., Dequen G. Deep Learning to Predict Hospitalization at Triage: Integration of Structured Data and Unstructured Text. Proceedings of the 2020 IEEE International Conference on Big Data (Big Data).

[47] Lovett P.B., Illg M.L., Sweeney B.E. (2016). A Successful Model for a Comprehensive Patient Flow Management Center at an Academic Health System. Am. J. Med. Qual..

[48] Kelen G.D., Wolfe R., D’Onofrio G., Mills A.M., Diercks D., Stern S.A., Wadman M.C., Sokolove P.E. (2021). Emergency Department Crowding: The Canary in the Health Care System. NEJM Catal. Innov. Care Deliv..

[49] Pollaris G., Sabbe M. (2016). Reverse Triage: More than Just Another Method. Eur. J. Emerg. Med..

[50] Syed S.T., Gerber B.S., Sharp L.K. (2013). Traveling Towards Disease: Transportation Barriers to Health Care Access. J. Community Health.

[51] McKenna P., Heslin S.M., Viccellio P., Mallon W.K., Hernandez C., Morley E.J. (2019). Emergency Department and Hospital Crowding: Causes, Consequences, and Cures. Clin. Exp. Emerg. Med..

[52] Boyle J., Jessup M., Crilly J., Green D., Lind J., Wallis M., Miller P., Fitzgerald G. (2012). Predicting Emergency Department Admissions. Emerg. Med. J..

[53] McManus M.L., Long M.C., Cooper A., Mandell J., Berwick D.M., Pagano M., Litvak E. (2003). Variability in Surgical Caseload and Access to Intensive Care Services. Anesthesiology.

[54] Litvak E., Fineberg H.V. (2013). Smoothing the Way to High Quality, Safety, and Economy. N. Engl. J. Med..

[55] Powell E.S., Khare R.K., Venkatesh A.K., Van Roo B.D., Adams J.G., Reinhardt G. (2012). The Relationship between Inpatient Discharge Timing and Emergency Department Boarding. J. Emerg. Med..

[56] Wong H.J., Wu R.C., Caesar M., Abrams H., Morra D. (2010). Smoothing Inpatient Discharges Decreases Emergency Department Congestion: A System Dynamics Simulation Model. Emerg. Med. J..

[57] Garson C., Hollander J.E., Rhodes K.V., Shofer F.S., Baxt W.G., Pines J.M. (2008). Emergency Department Patient Preferences for Boarding Locations When Hospitals Are at Full Capacity. Ann. Emerg. Med..

[58] Aaronson E.L., Yun B.J. (2020). Emergency Department Shifts and Decision to Admit: Is There a Lever to Pull to Address Crowding?. BMJ Qual. Saf..

[59] Ross M.A., Hockenberry J.M., Mutter R., Barrett M., Wheatley M., Pitts S.R. (2013). Protocol-Driven Emergency Department Observation Units Offer Savings, Shorter Stays, And Reduced Admissions. Health Aff..

[60] Al-Kuwaiti A., Hefny A.F., Bellou A., Eid H.O., Abu-Zidan F.M. (2012). Epidemiology of Head Injury in the United Arab Emirates. Turk. J. Trauma Emerg. Surg..

[61] Lim A.G., Kivlehan S., Losonczy L.I., Murthy S., Dippenaar E., Lowsby R., Yang M.L.C.L.C., Jaung M.S., Stephens P.A., Benzoni N. (2021). Critical Care Service Delivery across Healthcare Systems in Low-Income and Low-Middle-Income Countries: Protocol for a Systematic Review. BMJ Open.

